# Common procedures and conditions leading to inpatient hospital admissions in adults with and without diabetes from 2015 to 2019 in Germany: A comparison of frequency, length of hospital stay and complications

**DOI:** 10.1007/s00508-023-02153-z

**Published:** 2023-02-10

**Authors:** Alexander J. Eckert, Andreas Fritsche, Andrea Icks, Erhard Siegel, Annabel S. Mueller-Stierlin, Wolfram Karges, Joachim Rosenbauer, Marie Auzanneau, Reinhard W. Holl

**Affiliations:** 1 Institute of Epidemiology and Medical Biometry, ZIBMT, University of Ulm, Albert-Einstein-Allee 41, 89081 Ulm, Germany; 2 German Centre for Diabetes Research (DZD), Neuherberg, Germany; 3 Department of Internal Medicine, Division of Diabetology, Endocrinology and Nephrology, Eberhard-Karls University Tübingen, Tübingen, Germany; 4 Institute for Diabetes Research and Metabolic Diseases of the Helmholtz Centre Munich at the University of Tübingen, Tübingen, Germany; 5 Institute of Health Services Research and Health Economics, Centre for Health and Society, Medical Faculty, Heinrich-Heine-University Dusseldorf, Dusseldorf, Germany; 6 Institute for Health Services Research and Health Economics, German Diabetes Centre, Leibniz Centre for Diabetes Research at the Heinrich-Heine-University Dusseldorf, Dusseldorf, Germany; 7 Department of Gastroenterology, Diabetology, Endocrinology, and Nutritional Medicine, St. Josefskrankenhaus Heidelberg, Heidelberg, Germany; 8 Department of Psychiatry and Psychotherapy II, University Hospital Ulm, Ulm, Germany; 9 Division of Endocrinology and Diabetes, Medical Faculty, RWTH Aachen University, Aachen, Germany; 10 Institute for Biometrics and Epidemiology, German Diabetes Centre, Leibniz Centre for Diabetes Research at Heinrich Heine University Dusseldorf, Dusseldorf, Germany

**Keywords:** Hospitalization, Healthcare system, Billing data, Mortality, Orthopedics

## Abstract

**Objective:**

To evaluate common surgical procedures and admission causes in inpatient cases with diabetes in Germany between 2015 and 2019 and compare them to inpatient cases without diabetes.

**Methods:**

Based on the German diagnosis-related groups (G-DRG) statistics, regression models stratified by age groups and gender were used to calculate hospital admissions/100,000 individuals, hospital days as well as the proportion of complications and mortality in inpatient cases ≥ 40 years with or without a documented diagnosis of diabetes (type 1 or type 2).

**Results:**

A total of 14,222,326 (21%) of all inpatient cases aged ≥ 40 years had a diagnosis of diabetes. More middle-aged females with vs. without diabetes/100,000 individuals [95% CI] were observed, most pronounced in cases aged 40–< 50 years with myocardial infarction (305 [293–319] vs. 36 [36–37], *p* < 0.001). Higher proportions of complications and longer hospital stays were found for all procedures and morbidities in cases with diabetes.

**Conclusion:**

Earlier hospitalizations, longer hospital stays and more complications in inpatient cases with diabetes together with the predicted future increase in diabetes prevalence depict huge challenges for the German healthcare system. There is an urgent need for developing strategies to adequately care for patients with diabetes in hospital.

**Supplementary Information:**

The online version of this article (10.1007/s00508-023-02153-z) contains supplementary material, which is available to authorized users.

## Introduction

Approximately 8 million people with documented diabetes mellitus were living in Germany in 2020, implying a type 2 diabetes (T2D) prevalence of about 9% [1]. While the estimated number of undiagnosed cases decreased from around 2 million in 1997–1999 to 1.3 million in 2008–2011 [2], it has been estimated that the population with diagnosed diabetes will to rise to 12 million in 2040 according to data from statutory health insurances [1].

This development is not restricted to Germany but is predicted for several upper income and middle income countries, estimating a prevalence of up to 25% for diabetes for some of these countries in 2030 [3]. The increase in diabetes prevalence around the world [4] together with stable or slightly increasing per capita healthcare costs in individuals with diabetes in Germany (1.7 times higher than in individuals without diabetes) [5] could lead to challenging nationwide and global healthcare costs in the upcoming decades [6, 7].

Higher healthcare costs in people with diabetes are mainly the consequence of prescribed medication from pharmacies and inpatient treatment [5] but also of outpatient treatment and indirect costs, e.g., due to reduced productivity in the work place [8]. Higher inpatient costs in patients with diabetes may be due to more frequent hospitalizations, longer hospital stays or more complications compared to people without diabetes. There are few publications reporting a high prevalence of diabetes among hospitalized cases [9] as well as frequent readmissions in people with diabetes [10] but data on admission rates (accounting for the respective reference population with and without diabetes) are scarce. Furthermore, it is less clear which procedures and diagnoses are mostly related to more frequent hospital admissions, longer hospital stays as well as higher rates of complications and mortality among individuals with type 1 diabetes (T1D) or T2D compared to those without diabetes.

The aim of this study was to compare the frequency and outcomes of inpatient hospital admissions for several high-volume procedures and diagnoses between all cases with or without diabetes from 2015 to 2019 on a nationwide basis using mandatorily documented data in Germany.

## Patients, material and methods

### Data source and participants

Data were obtained from the diagnosis-related groups (DRG) statistics, collected yearly by the German Federal Statistical Office (Statistisches Bundesamt, DESTATIS) since 2004. All general hospitals are required to send annual data on all inpatient services to the Institute for the Hospital Remuneration System (Institut für das Entgeltsystem im Krankenhaus, InEK). The InEK then sends a legally defined list of parameters to DESTATIS. The DRG statistics therefore include case-related data from all German hospitals based on §1 of the Hospital Remuneration Act (Krankenhausentgeltgesetz, KHEntgG) [11].

The analysis of the most recently available 5 years before the coronavirus disease 2019 (COVID-19) pandemic of the DRG statistics (source: Research Data Center, Forschungsdatenzentrum, FDZ, of the German Federal and State Statistical Offices, DRG statistics 2015–2019) was performed via controlled remote data processing. For data protection reasons, the data are structured by treatment case and not by patient: repeated admissions of the same patient can therefore not be aggregated. The analysis programs were created using SAS 9.4 (Statistical Analysis Software, SAS Institute, Cary, NC, USA) and sent to the FDZ. Results were released by the FDZ following the disclosure and clearance of the results.

**Fig. 1:**
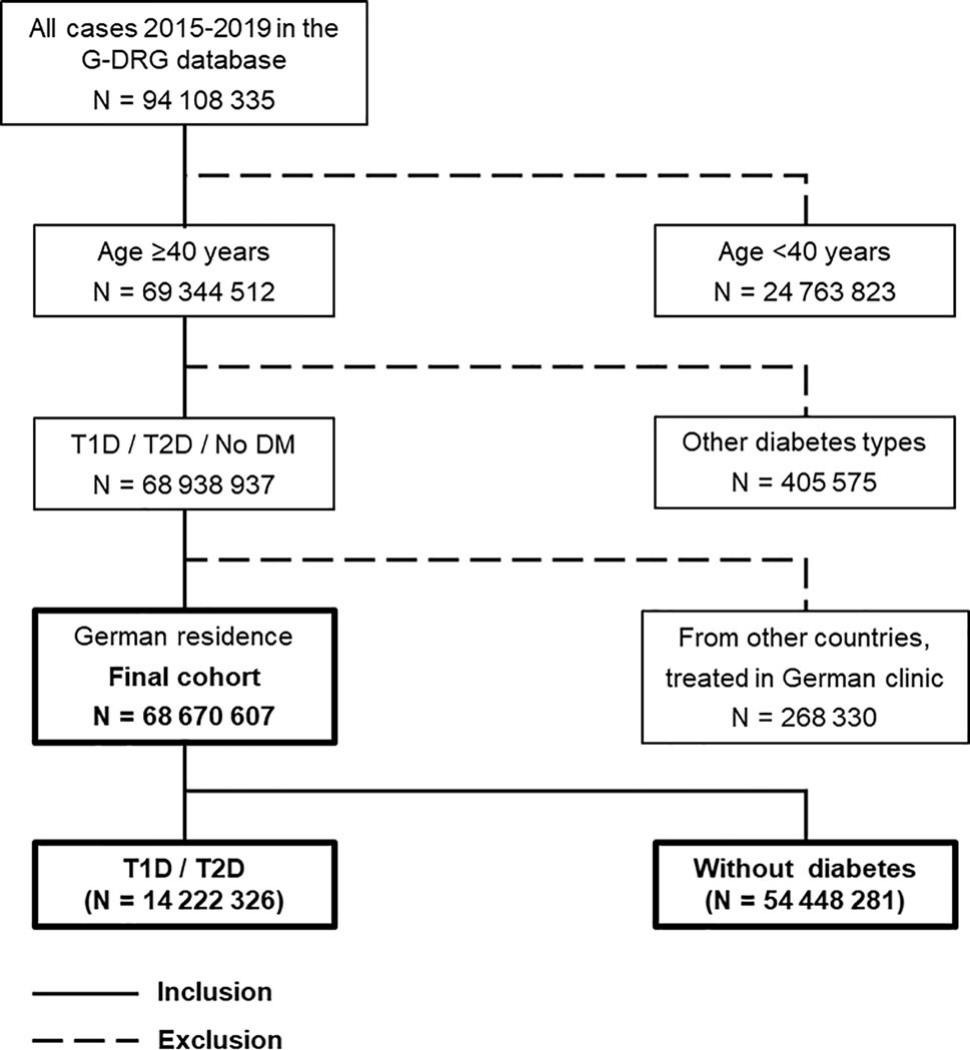
Included and excluded cases from the G‑DRG database in 2015–2019. *T1D *type 1 diabetes, *T2D* type 2 diabetes, *No DM* no diabetes, *Other diabetes types *gestational diabetes, pancreatic diabetes, rare types of diabetes (ICD-10 E12, E14, prediabetes)

All German inpatient cases from 2015–2019 aged ≥ 40 years without or with encoded T1D or T2D were included. Case identification was assessed according to ICD-10 coding as main or secondary diagnosis (E10 for T1D, E11 for T2D). Cases with other diabetes diagnoses or unclear diagnoses were excluded. Cases from other countries, engaging the healthcare service of German hospitals (*n* = 268,330) were excluded as well. Cases with unknown gender (*n* = 3376/68,670,607, representing 0.005%) were assigned to the female group which was the larger group. Figure 1 shows included and excluded cases.

### Relevant procedures and outcome variables

Classification of procedures and diagnoses was according to the German operations and procedures classification (Operationen- und Prozedurenschlüssel, OPS) versions and to the ICD-10-GM (German modification) versions of the respective reporting year. We decided to investigate procedures and diagnoses that are frequent in German hospitals, well defined and encompass different medical specialities to provide a broad overview of the inpatient care in Germany. Therefore, we analyzed initial hip (OPS 5‑820) and knee endoprosthis (OPS 5‑822), spine surgery (OPS 5‑83), shoulder refixations (OPS 5‑814), appendectomy (open or laparoscopic, OPS 5‑470) and cholecystectomy (OPS 5‑511) as well as acute myocardial infarction (ICD-10 I21) and stroke (ICD-10 I63). We investigated the frequency of these cases, the length of hospital stay (days), the proportion with complications (supplementary table 1 provides all ICD-10 codes that were used to define inpatient complications as primary or secondary diagnosis) and the mortality ratio between cases with and without diabetes (T1D and T2D combined).

### Population reference data

For frequency analyses of procedures and diagnoses, we calculated the proportion of aggregated cases per 100,000 individuals of the respective aggregated populations from 2015–2019 (with or without diabetes). Data for the whole German population of these years were taken from DESTATIS [12].

The population with T1D was estimated from T1D prevalence estimates from the Robert Koch Institute (RKI) Diabetes Surveillance Report which used data from the German prospective diabetes patient follow-up registry (DPV), from the North Rhine-Westphalian diabetes registry [13], and total population data.

The population with T2D diabetes was estimated from total population data and T2D prevalence estimates from the Central Research Institute of Ambulatory Health Care in Germany (Zentralinstitut für die kassenärztliche Versorgung, ZI) derived from the nationwide billing data of panel doctors for 2015 [14] and the population size on 31 December 2017, which is estimated based on the 2011 census data [12].

In the prevalence estimates of the Central Institute for Statutory Health Care, all patients with the confirmed main or secondary diagnoses E11, E14 (not otherwise specified diabetes mellitus) or unclear diabetes mellitus (with different coding) in at least two quarters of the year were allocated to type 2 diabetes. These estimates based on nationwide billing data of panel doctors exclude approximately 13.9% of the population (including, but not limited to, members of private health insurances) [14].

Because of the lower numbers of DRG cases and individuals with T1D, especially in higher age groups and the fact that population data for individuals with diabetes can only be estimated and not be measured exactly, we decided to combine T1D and T2D and excluded rarer diabetes forms instead of analyzing diabetes types separately. Therefore, only cases with diabetes were compared to cases without diabetes.

### Statistical analysis

For the analysis of proportions of persons with any procedure or diagnosis as well as proportions of people with a specific procedure/diagnosis, we performed unadjusted logistic regression models with diabetes (yes/no) as independent variable stratified by gender and age groups (40–< 50 years, 50–< 60 years, 60–< 70 years, 70–< 80 years, ≥ 80 years). The respective cumulative cases in 2015–2019 divided by the aggregated population for 2015–2019 (reported per 100,000 individuals) were used as dependent variable. Linear regression models were used to calculate length of hospital stay (days) and logistic regression models were performed for the proportion of hospitalized persons with a specific procedure or diagnosis with incurring complications/fatal consequences, in each case stratified by gender and age groups and with diabetes (yes/no) as independent variable. All *p*-values were adjusted for multiplicity using the Tukey-Kramer method. Due to the large number of cases included, significance was considered as *p* < 0.01. All outcomes were presented in graphs showing the calculated values per age group, stratified by gender. For better visibility, these values were connected with smoothed spline curves via SigmaPlot (Systat Software Inc, San Jose, CA, USA), Version 13.0.

## Results

### Study population

Between 2015 and 2019, the average annual population ≥ 40 years of age in Germany was 47,133,407 and a total of 68,670,607 inpatient cases of the same age from the DRG database were registered. Of the inpatient cases 21% (14,222,326) had T1D or T2D documented as principal or secondary diagnosis, while an estimated 15% (7,155,570) of the total German population ≥ 40 years of age had T1D or T2D. Overall, 49.5% of inpatient cases were male with median age [lower and upper quartiles] of 70 [58; 79] years, whereas 47.6% of the total population were male. The proportion of males was 54.3% in inpatient cases with diabetes with median age 75 [66; 81] years, and 57.9% of the population with diabetes were male. The number of total cases, cases for each procedure or diagnosis and population data stratified by age groups, gender, and diabetes (yes/no) are presented in Table 1.

**Table 1:** Total number of cases with all evaluated procedures and diagnoses, and population at risk, stratified by age group, gender and diabetes

Cases	Cases with diabetes by age group (years)					Cases without diabetes by age group (years)				
	40–< 50	50–< 60	60–< 70	70–< 80	≥ 80	40–< 50	50–< 60	60–< 70	70–< 80	≥ 80
*All*										
Population (2015–2019)^a^	2,210,075	5,852,567	9,089,704	10,632,125	7,993,381	52,588,086	60,270,687	40,512,338	29,187,761	17,330,311
All inpatient cases	447,544	1,496,586	2,999,197	4,847,710	4,431,289	6,556,192	10,659,425	11,190,623	13,586,854	12,455,187
Hip replacement	1679	11,835	36,595	69,591	64,028	34,534	134,458	235,563	332,703	262,498
Knee replacement	1988	17,979	48,707	64,314	20,106	21,965	129,255	223,336	269,883	93,984
Spine surgery	8275	30,622	55,498	84,019	41,560	187,943	281,555	254,074	315,157	163,835
Shoulder refixation	4834	17,125	19,173	11,672	2003	84,947	172,435	111,706	56,658	9172
Appendectomy	2955	10,017	21,301	28,797	13,446	81,425	116,388	111,946	104,387	44,785
Cholecystectomy	6312	18,300	33,371	44,254	27,208	132,810	187,552	164,177	144,514	79,395
Myocardial infarction	9886	37,543	68,870	103,113	91,330	53,337	143,471	159,691	189,654	193,507
Stroke	5681	27,290	66,149	123,196	135,059	36,115	99,729	151,487	255,774	342,951
*Male*										
Population (2015–2019)^a^	1,510,859	4,220,477	5,979,833	5,840,731	3,152,765	26,130,843	28,978,382	18,017,365	12,264,473	6,137,308
All inpatient cases	262,799	949,397	1,879,237	2,740,221	1,888,929	3,167,029	5,631,534	5,947,345	6,666,279	4,879,617
Hip replacement	1021	7152	19,684	30,184	20,082	18,562	67,890	99,856	116,790	75,763
Knee replacement	842	8238	21,527	27,439	7474	8738	54,951	88,244	98,280	30,383
Spine surgery	4331	18,567	31,375	41,184	16,927	86,006	149,264	126,895	128,882	56,976
Shoulder refixation	2782	11,098	12,036	6819	1138	45,698	94,999	62,090	28,703	4614
Appendectomy	1704	6324	13,756	17,578	7121	41,252	60,678	61,062	55,017	21,800
Cholecystectomy	2675	9128	18,330	25,147	13,132	46,625	71,162	67,127	67,518	35,240
Myocardial infarction	7750	29,674	51,062	67,098	45,536	43,693	116,454	119,190	122,799	95,675
Stroke	3761	19,852	45,659	71,994	55,129	21,688	68,292	97,476	136,248	125,870
*Female*										
Population (2015–2019)^a^	699,216	1,632,090	3,109,871	4,791,394	4,840,616	26,457,243	31,292,305	22,494,973	16,923,288	11,193,003
All inpatient cases	184,745	547,189	1,119,960	2,107,489	2,542,360	3,389,163	5,027,891	5,243,278	6,920,575	7,575,570
Hip replacement	658	4683	16,911	39,407	43,946	15,972	66,568	135,707	215,913	186,735
Knee replacement	1146	9741	27,180	36,875	12,632	13,227	74,304	135,092	171,603	63,601
Spine surgery	3944	12,055	24,123	42,835	24,633	101,937	132,291	127,179	186,275	106,859
Shoulder refixation	2052	6027	7137	4853	865	39,249	77,436	49,616	27,955	4558
Appendectomy	1251	3693	7545	11,219	6325	40,173	55,710	50,884	49,370	22,985
Cholecystectomy	3637	9172	15,041	19,107	14,076	86,185	116,390	97,050	76,996	44,155
Myocardial infarction	2136	7869	17,808	36,015	45,794	9644	27,017	40,501	66,855	97,832
Stroke	1920	7438	20,490	51,202	79,930	14,427	31,437	54,011	119,526	217,081

^a^ Data for the whole German population of the years 2015–2019 were taken from DESTATIS [11]. The population with T1D was based on estimations from the Robert Koch Institute (RKI) Surveillance Report^12^, the population with T2D was based on the estimated T2D prevalence from the Central Research Institute of Ambulatory Health Care in Germany (Zentralinstitut für die kassenärztliche Versorgung, Zi) by reference to the nationwide billing data of panel doctors for 2015^13^

### Results for any procedure or diagnosis among the whole study population

**Fig. 2:**
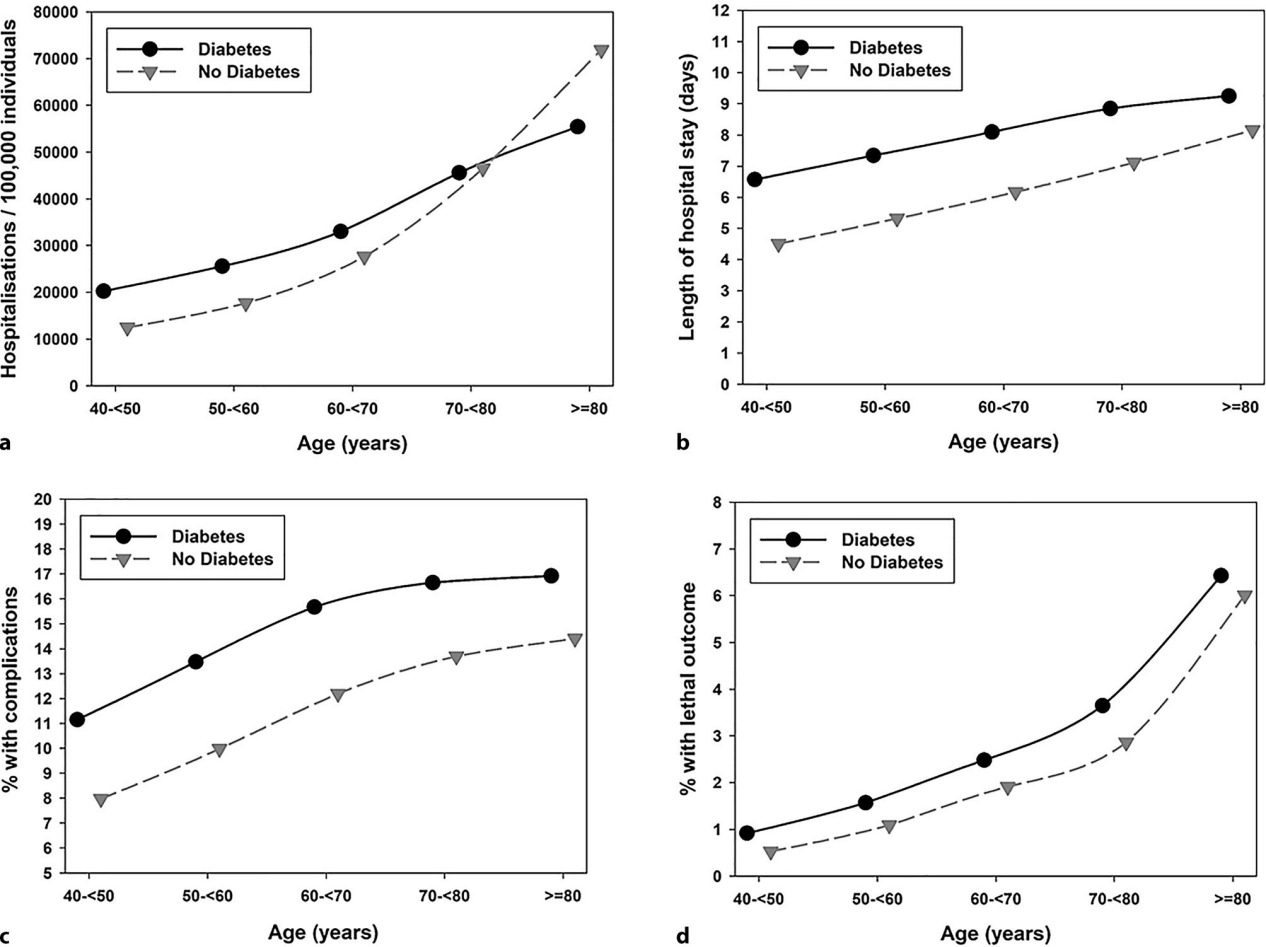
Frequency of hospitalizations/100,000 individuals (**a**), length of hospital stays (**b**), rate of complications (**c**) and rate of mortality (**d**) among all hospitalized patients with and without diabetes in Germany from 2015 to 2019

Overall, the number of aggregated inpatient cases/100,000 individuals (of the aggregated German population 2015–2019) increased with higher age for both groups with and without diabetes. The number of inpatient cases/100,000 individuals was higher in the population with diabetes vs. no diabetes for the age group 40–< 60 years, but lower in those aged ≥ 80 years. Length of hospital stay as well as the proportion with complications and the mortality rate was increased in inpatient cases with diabetes over all age groups (Fig. 2).

### Results stratified for age groups and gender and for specific procedures or diagnoses

#### Hospitalization rates

**Fig. 3:**
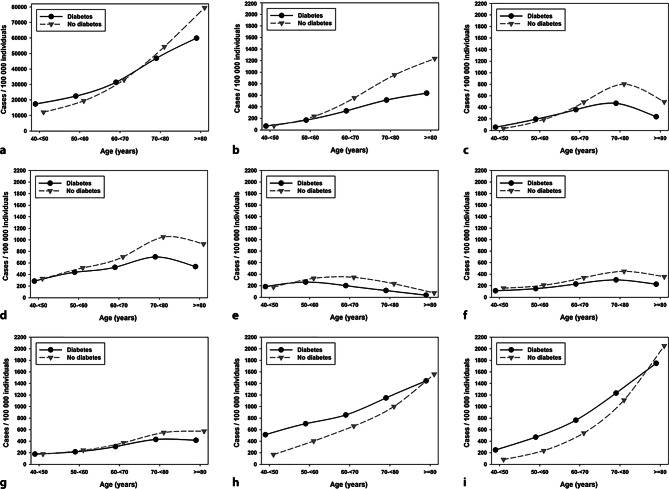
Frequency of hospitalization (**a**) and procedures and diagnoses/100,000 individuals **b**–**i** among all hospitalized men with and without diabetes in Germany from 2015 to 2019. **a** Frequency of hospitalization, **b** hip replacement, **c** knee replacement, **d** spine surgery, **e** shoulder refixation, **f** appendectomy, **g** cholecystectomy, **h** myocardial infarction, **i** stroke

In males, the number of cases/100,000 individuals was similar between the populations with and without diabetes for all surgeries at the age of 40–< 60 years and more frequent in the population without diabetes in higher age groups (Fig. 3b–g). Hospitalization for myocardial infarction and stroke was significantly (all *p* < 0.001) more frequent in males with vs. without diabetes up to the age of 80 years. Especially in the youngest age group, the proportion [95%-confidence interval] of myocardial infarction (513 [502–524] vs. 167 [166–168] cases/100,000 individuals) and stroke (249 [241–257] vs. 83 [82–84] cases/100,000 individuals) was tripled in the population with diabetes (Fig. 3h, i).

**Fig. 4:**
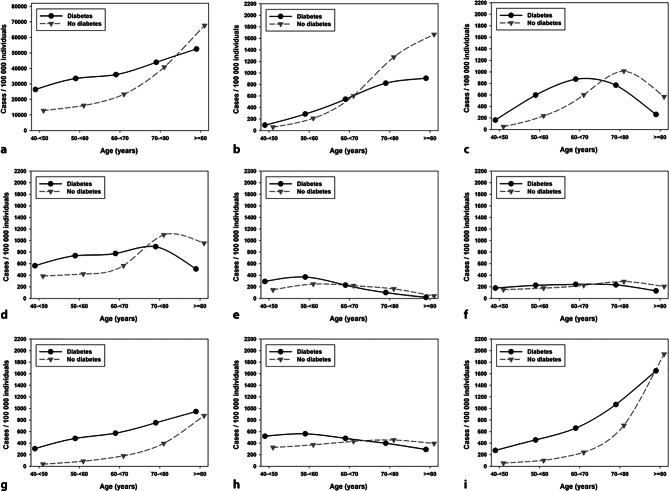
Frequency of hospitalization (**a**) and procedures and diagnoses/100,000 individuals **b**–**i** among all hospitalized women with and without diabetes in Germany from 2015 to 2019. **a** Frequency of hospitalization, **b** hip replacement, **c** knee replacement, **d** spine surgery, **e** shoulder refixation, **f** appendectomy, **g** cholecystectomy, **h** myocardial infarction, **i** stroke

In females, the number of hospitalized cases/100,000 individuals in the population was significantly (all *p* < 0.001) higher for cases with diabetes up to the age of 60 years regarding hip replacement and shoulder refixation, until the age of 70 years regarding knee replacement, spine surgery, appendectomy and cholecystectomy, until the age of 80 years for stroke, and through all age groups for myocardial infarction. With higher age these cases were more frequent among the population without diabetes (Fig. 4b–i). The most remarkable difference with a more than 8‑fold higher admission rate in the population with diabetes was observed for myocardial infarction in females aged 40–< 50 years (305 [293–319] vs. 36 [36–37] cases/100,000 individuals), followed by stroke (275 [262–287] vs. 55 [54–55] cases/100,000 individuals, *p* < 0.001) and knee replacement (164 [155–174] vs. 50 [49–51] cases/100,000 individuals, *p* < 0.001).

#### Length of hospital stay

Length of hospital stay was significantly increased in males and females with vs. without diabetes for all hospitalized cases (supplementary figures 1A and 2A) and throughout all procedures and diagnoses analyzed (supplementary figures 1B–I and 2B–I) except shoulder refixation and knee replacement in those aged 40–< 50 years. Differences in length of hospital stay ranged from 1 to 3 additional days for most procedures and diagnoses and results were similar for males and females. Highest differences of hospital days [95% confidence interval, CI] between inpatient cases with vs. without diabetes could be observed for appendectomy (11.3 [10.7–11.9] vs. 6.3 [6.2–6.5] days in males and 10.5 [9.8–11.2] vs. 6.0 [5.9–6.1] days in females, all *p* < 0.001) in the age group of 40–< 50 years (supplementary figures 1F and 2F).

#### Complications and mortality

The proportion of complications was elevated for nearly all procedures and diagnoses in inpatient cases with versus without diabetes over all age groups in men (supplementary figure 3A–I) and women (supplementary figure 4A–I). The most remarkable differences with nearly doubled proportion of complications in inpatient cases with vs. without diabetes were observed for appendectomy in the age group of 40–< 50 years (18.0 [16.2–19.9] vs. 9.2 [9.2–9.5] % in males and 19.8 [17.7–22.1] vs. 9.6 [9.3–9.9] % in females, all *p* < 0.001, figures 3F and 4F) and for cholecystectomy in male inpatient cases aged 40–< 50 years (15.7 [14.3–17.1] vs. 8.5 [8.3–8.8] %, *p* < 0.001, Fig. 3g). Only in the age groups of 40–< 50 years were some differences for complication rates not significant, such as for males (*p* = 0.999) and females (*p* = 0.999) with knee replacement as well as hip replacement (*p* = 0.518), myocardial infarction (*p* = 0.398) and stroke (*p* = 0.058) in female inpatient cases. Shoulder refixation revealed no significant differences for complications between inpatient cases with and without diabetes below the age of 60 years (supplementary figures 3E and 4E).

The inpatient mortality increased with higher age for all procedures and diagnoses for inpatient cases with and without diabetes. The most prominent differences in mortality between inpatient cases with vs. without diabetes were detected at the age of 40–< 50 years for appendectomy (5.8 [4.8–7.0] vs. 1.4 [1.3–1.5] % in males and 5.9 [4.7–7.4] vs. 0.9 [0.8–1.0] % in females, all *p* < 0.001, Figs. 3f and 4f). The mortality in spine surgery was increased in inpatient cases with diabetes over all age groups with the highest ratio between inpatient cases with vs. without diabetes at the age of 40–< 50 years (0.85 [0.62–1.18] vs. 0.18 [0.16–0.21] % in males and 0.43 [0.27–0.69] vs. 0.12 [0.10–0.14] % in females, all *p* < 0.001, supplementary figures 3D and 4D). For knee replacement and shoulder refixation there were hardly any cases with fatal consequences in younger age groups and therefore we excluded the age group of 40–< 50 years from this specific analysis. Significant differences were only visible in male inpatient cases with shoulder refixation or knee replacement aged 70 years or higher (all *p* < 0.01) and female inpatient cases with knee replacement aged 60–< 70 years or ≥ 80 years (all *p* < 0.001). Similar results were observed for hip replacement where significant differences in mortality were detected only in inpatient cases aged 60 years or higher (all *p* < 0.001). No remarkable difference between inpatient cases with diabetes and controls could be observed for myocardial infarction and stroke, except a higher mortality in inpatient cases without diabetes aged ≥ 80 years for myocardial infarction and in female inpatient cases with stroke (supplementary figures 3H–I and 4H–I).

## Discussion

This is the largest evaluation in Germany of data on frequency of hospitalization, length of hospital stays and complications in more than 14 million inpatient cases with diabetes compared to more than 54 million inpatient cases without diabetes between the years 2015 and 2019. Every fifth inpatient case aged over 40 years in Germany had a diagnosis of type 1 or type 2 diabetes. We could detect higher hospitalization rates in the population with diabetes compared to without diabetes. Higher proportion of complications as well as longer hospital stay were observed in inpatient cases with vs. without diabetes for nearly all procedures and diagnoses over all age groups. Mortality was generally higher in inpatient cases with vs. without diabetes.

Data on the frequency of initial inpatient admission rates referring to the respective population are scarce; however, higher hospital readmission rates in people with diabetes have been reported previously [10]. The risks for revision of hip endoprosthesis [15] as well as readmission because of cardiac diagnoses [16, 17] have particularly been mentioned in the literature. More frequent hospital admissions in individuals with diabetes have been reported for appendectomy [18] but not for cholecystectomy [19]. We found a higher hospitalization rate for nearly all procedures and diagnoses in females aged 40 up to 70 years (magnitude depending on the procedure). In males these findings were restricted to myocardial infarction and stroke. We are not aware of previous studies reporting such gender differences regarding hospitalization for surgery; however, it is known that male individuals are diagnosed with diabetes earlier than females [20] leading to a higher prevalence of diabetes in middle-aged men than women, but the impact of diabetes on mortality is stronger in females [21]. As we analyzed case-related data, we could not ascertain whether the differences in hospitalization rates between females with and without diabetes were mainly because of a higher risk for inpatient admission in general or due to readmissions of some individuals, except for hip and knee replacements where revisions are separately encoded (hip: OPS 5‑821, knee: OPS 5‑823). It is assumed that readmissions are one of the reasons for higher hospitalization rates of people with diabetes concerning myocardial infarction and stroke. Diabetes was considered as risk factor for readmission 30 days after myocardial infarction [17] and as a possible risk factor for readmissions after stroke according to a systematic review but data were too heterogeneous to provide clear evidence [16]. We assume that both risk for initial hospital admissions and readmissions are responsible for the higher number of inpatient cases/100,000 individuals in the population with diabetes, especially in females. Additionally, there seems to be a shift towards earlier orthopedic surgery in the population with diabetes. The higher frequency of orthopedic procedures in individuals without diabetes in higher age groups could therefore be due to the fact that the surgery was already conducted in earlier ages in people with diabetes. Furthermore, the admission rate for individuals with diabetes might be still underestimated, because of undiagnosed inpatient cases of diabetes. A survey to estimate the prevalence of T2D among patients aged ≥ 55 years in German hospitals found a proportion of 9.5% of individuals with unrecognized T2D at admission [22]. Another study reported a rate of 4% with undiagnosed diabetes in hospitalized patients aged 50 years or older [23]. It must be kept in mind that in many hospitalizations for diabetes, the diagnosis of diabetes might not be documented, especially because in the German payment system (DRG), diabetes yields no high return compared to other diagnoses. Furthermore, we used billing data of panel doctors, which excludes about 14% of the population (especially from private health insurances) to estimate the proportion with T2D of the entire population, but the DRG statistics cover the whole German population. Consequently, the real prevalent population with type 2 diabetes may differ slightly, which, in turn may have led to a misjudgement of the proportion of inpatient cases among patients with diabetes. Furthermore, it must be assumed that the prevalence of T2D has increased since 2011 which would have led to an underestimation of the actual T2D population in the years 2015–2019. Considering all these limitations, the total admission rate must be interpreted with caution, but the shift towards younger ages and the gender differences in individuals with diabetes remains as all these limitations should be equipollent in all subgroups. Especially the lower rate of surgery in men with diabetes needs to be observed in future as it might depict a possible undertreatment among this group.

Data from the United Kingdom (UK) Arthroplasty Pain Experience (APEX) trials were similar to our results on length of hospital stay in knee and hip replacements with about 1 day longer stays in individuals with diabetes; however, the overall length of hospital stay was nearly doubled in our cohort and the differences in hospital days in the APEX study vanished after adjustment for further comorbidities [24]. This might suggest that longer hospital stays in inpatient cases with diabetes might be a consequence of the combination of diabetes itself and diabetes-associated long-term complications and comorbidity. In addition, hospital acquired complications were markedly higher in diabetes patients, which also accounts for longer hospital stays. It is further known that in Germany hospital stays are generally longer than in most other European countries [25], which can be partly explained by the German healthcare system that provides three times more hospital beds than the UK [26] (7.9 vs. 2.4 per 1000 inhabitants). In addition, diabetes treatment initiated and adjusted during a hospital stay for a surgical procedure may add to the excess length of hospitalization in cases with diabetes. Spine surgery was mentioned in a previous review to be associated with longer hospital stays, more complications, higher mortality and higher risk for readmissions in individuals with diabetes [27], which is in line with our results. Another study found that the length of stay is highly dependent on glycemic control. The authors reported a difference of up to 5 days in hospital stay between people with uncontrolled diabetes and individuals without diabetes, but only 1 day difference in patients with controlled diabetes [28]. These are important results suggesting that the risk for longer hospital stays in people with diabetes might be markedly reducible by improving glycemic control. We found especially high differences in the length of hospital stays for appendectomy comparing cases with and without diabetes aged 40–< 60 years. Longer hospital stay for appendectomy was previously mentioned for individuals with diabetes [29, 30] and in people with preoperative fasting blood glucose levels of ≥ 123 mg/dl [31]. Length of hospital stay and healthcare costs were lower in patients with diabetes for laparoscopic appendectomy compared to open appendectomy according to a study from Taiwan. The authors proposed that laparoscopic appendectomy should be used particularly in individuals with diabetes to reduce the risk for longer hospital stay and healthcare costs [32]. Our results depict that this might be especially important in middle-aged people (40–< 50 years) with diabetes, where length of stay and the proportion of complications were nearly doubled compared to cases without diabetes.

It must be kept in mind that especially in the younger age groups other factors besides the diabetes itself might contribute to the higher number of orthopedic surgeries and the longer hospital stay. Obesity and reduced physical activity could be both underlying reasons for premature type 2 diabetes and risk factors for orthopedic surgery as well as for longer hospital stay, but the extent is still discussed in the literature [33–35].

We detected higher mortality rates in cases with diabetes compared to without diabetes for most procedures. Appendectomy, cholecystectomy and spine surgery were procedures with increased mortality in patients with diabetes over all age groups. In terms of appendectomy and cholecystectomy, this is in line with previous studies [36, 37], while data on mortality in cases with diabetes undergoing spine surgeries are controversial [38, 39]. Our results indicate that spine surgery in individuals with diabetes should get high attention in hospitals, because despite the overall low mortality rate in this procedure, inpatient cases with diabetes showed an up to 4‑fold higher risk for fatal outcome compared to inpatient cases without diabetes. For hip and knee endoprostheses as well as shoulder surgery, our findings are quite congruent to the literature concerning complications in diabetes patients. Infections, the risk for revision of endoprosthesis or tendon re-tearing at the shoulder joint, are often reported [40–43]. Publications on mortality are scarce for these procedures, which might be due to the overall low mortality associated with these surgeries. We found higher mortality in cases with diabetes for hip and knee replacements and for shoulder refixation but only above the age of 70 years.

The strength of this study was the coverage of all inpatient cases between 2015 and 2019 in Germany irrespective of their insurance status, providing a representative picture of the actual hospitalizations and complications for inpatient cases with and without diabetes. Limitations were that the DRG data are only case-related and therefore no information on the patient level was obtainable. Additionally, it must be mentioned that the cases are based on billing data which could have influenced the coding of diagnoses and the classification of diabetes types to some extent. For this reason, we decided to combine type 1 and type 2 diabetes and exclude other rarer diabetes types. In addition, we cannot exclude that some cases may have had unrecognized diabetes. The results for hospital admissions/100,000 individuals must further be interpreted with caution as the prevalence of T1D and T2D is only an estimation based on data from previous years, while data on the whole German population are assumed to be relatively precise. Therefore, we concentrated on remarkable differences in hospitalization rates and age distribution. Furthermore, due to the structure of case-related billing data with restricted variable content, additional information on the diabetes disease, such as duration of disease, glycemic control, diabetes-related comorbidities and medication could not be analyzed. The same applies to relevant patient characteristics, namely social background, educational status and ethnicity.

The longer hospital stays as well as higher proportion of complications and mortality in patients with compared to those without diabetes clearly indicate that inpatient care of these patients must be intensified, especially in departments not specialized for the treatment of diabetes. Therefore, trained consultant diabetes specialists are desirable in hospitals. Furthermore, higher hospitalization rates in younger patients with diabetes especially for orthopedic surgery point at an increased disease burden in patients with diabetes. Lower hospitalization rates for orthopedic procedures in patients with diabetes in the high age groups might indicated that surgical treatment in these high-risk patients is avoided.

As the prevalence of diabetes is likely to increase in the next decades and individuals with diabetes need surgery earlier, have longer hospital stays, more complications and higher mortality than the general population, these results depict a challenging future for the German healthcare system. People living with diabetes requiring surgery represent a vulnerable group, and sufficient personnel trained in diabetes care is required in all hospitals. In addition, it is important to lower the rate of unrecognized diabetes by screening prior to hospital admission, to enable adequate medical care. Despite the fact that hospitalization rates and length of stay differ between healthcare systems, our results should be transferable to many middle income and high income countries.

## Supplementary Information

Supplementary table 1. ICD-10 codes and diagnoses that were combined as “complications”.

Supplementary figure 1. Length of hospital stays of all hospitalizations (A) and several procedures and diagnoses (B)–(I) among all hospitalized men with and without diabetes in Germany from 2015 to 2019.

Supplementary figure 2. Length of hospital stays of all hospitalizations (A) and several procedures and diagnoses (B)–(I) among all hospitalized women with and without diabetes in Germany from 2015 to 2019.

Supplementary figure 3. Complication and mortality rates of all hospitalizations (A) and several procedures and diagnoses (B)–(I) among all hospitalized men with and without diabetes in Germany from 2015 to 2019.

Supplementary figure 4. Complication and mortality rates of all hospitalizations (A) and several procedures and diagnoses (B)–(I) among all hospitalized women with and without diabetes in Germany from 2015 to 2019.
