# Supplementary figures and images for: Common procedures and conditions leading to inpatient hospital admissions in adults with and without diabetes from 2015 to 2019 in Germany: A comparison of frequency, length of hospital stay and complications
Source: Wien Klin Wochenschr. 2023 Feb 10;135(13-14):325–35. doi: 10.1007/s00508-023-02153-z (PMC9913003; doi:10.1007/s00508-023-02153-z)

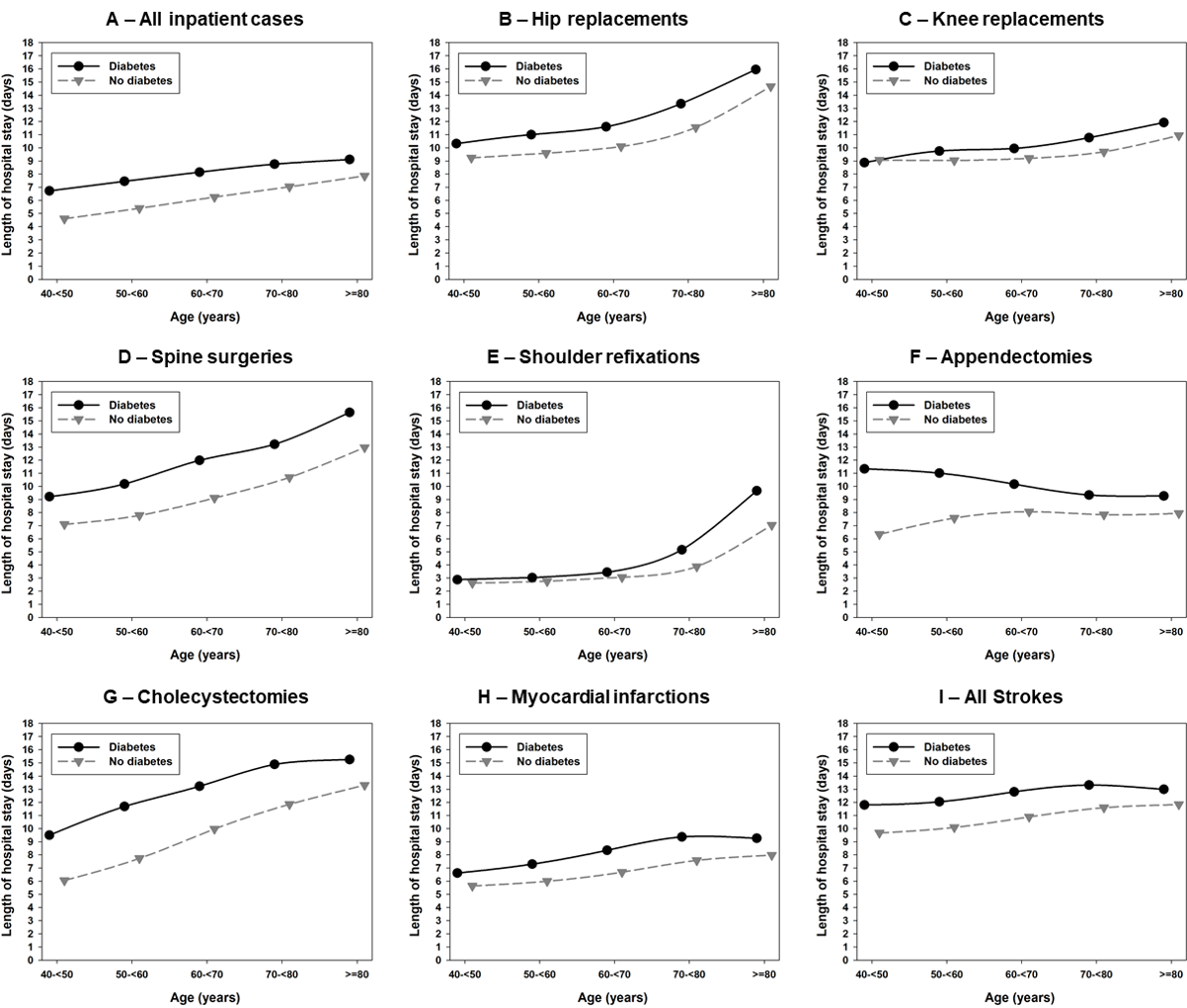

Supplement: Supplementary file 2 — Supplementary figure 1. Length of hospital stays of all hospitalizations (A) and several procedures and diagnoses (B)–(I) among all hospitalized men with and without diabetes in Germany from 2015 to 2019. [file 508_2023_2153_MOESM2_ESM.tif]

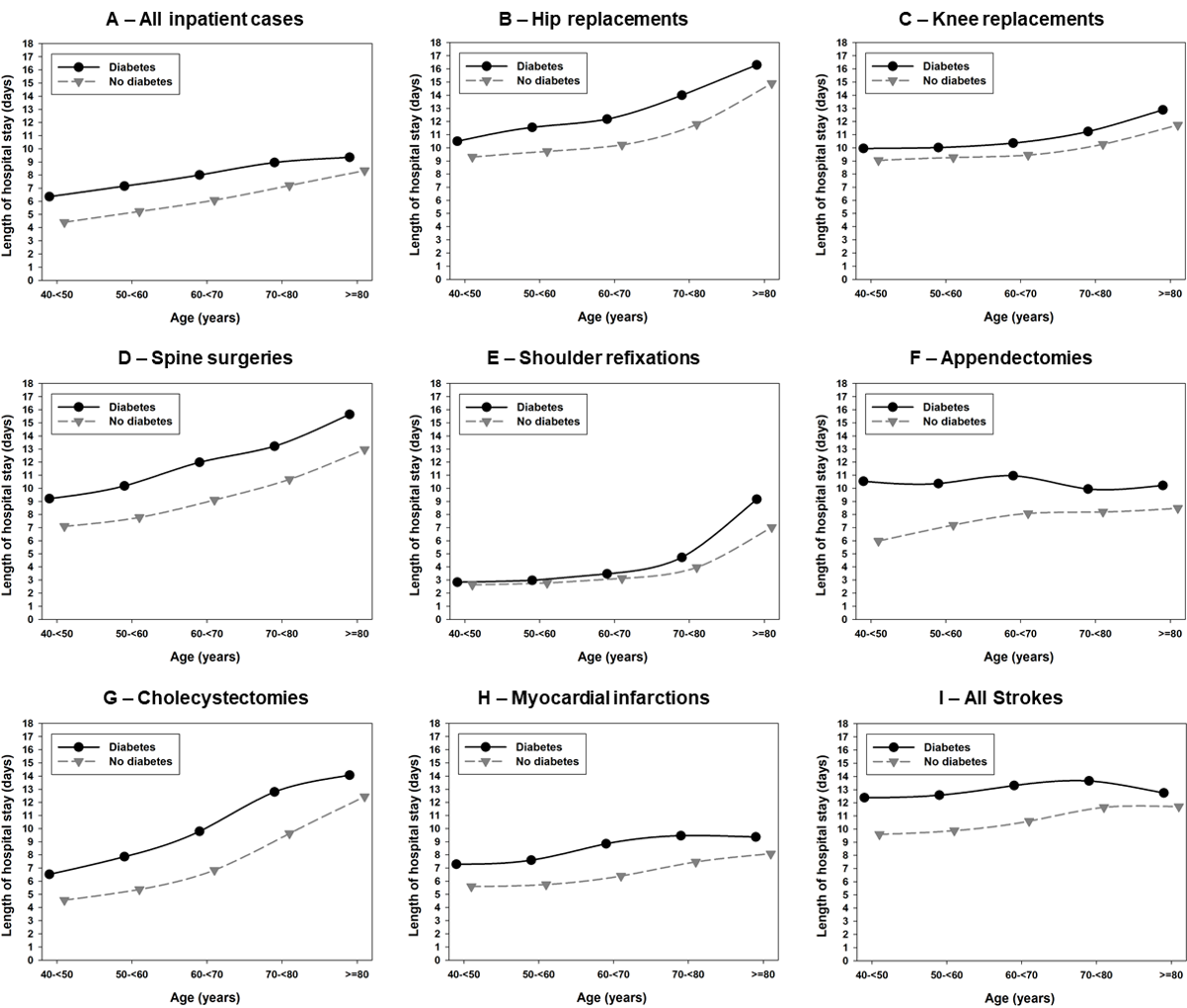

Supplement: Supplementary file 3 — Supplementary figure 2. Length of hospital stays of all hospitalizations (A) and several procedures and diagnoses (B)–(I) among all hospitalized women with and without diabetes in Germany from 2015 to 2019. [file 508_2023_2153_MOESM3_ESM.tif]

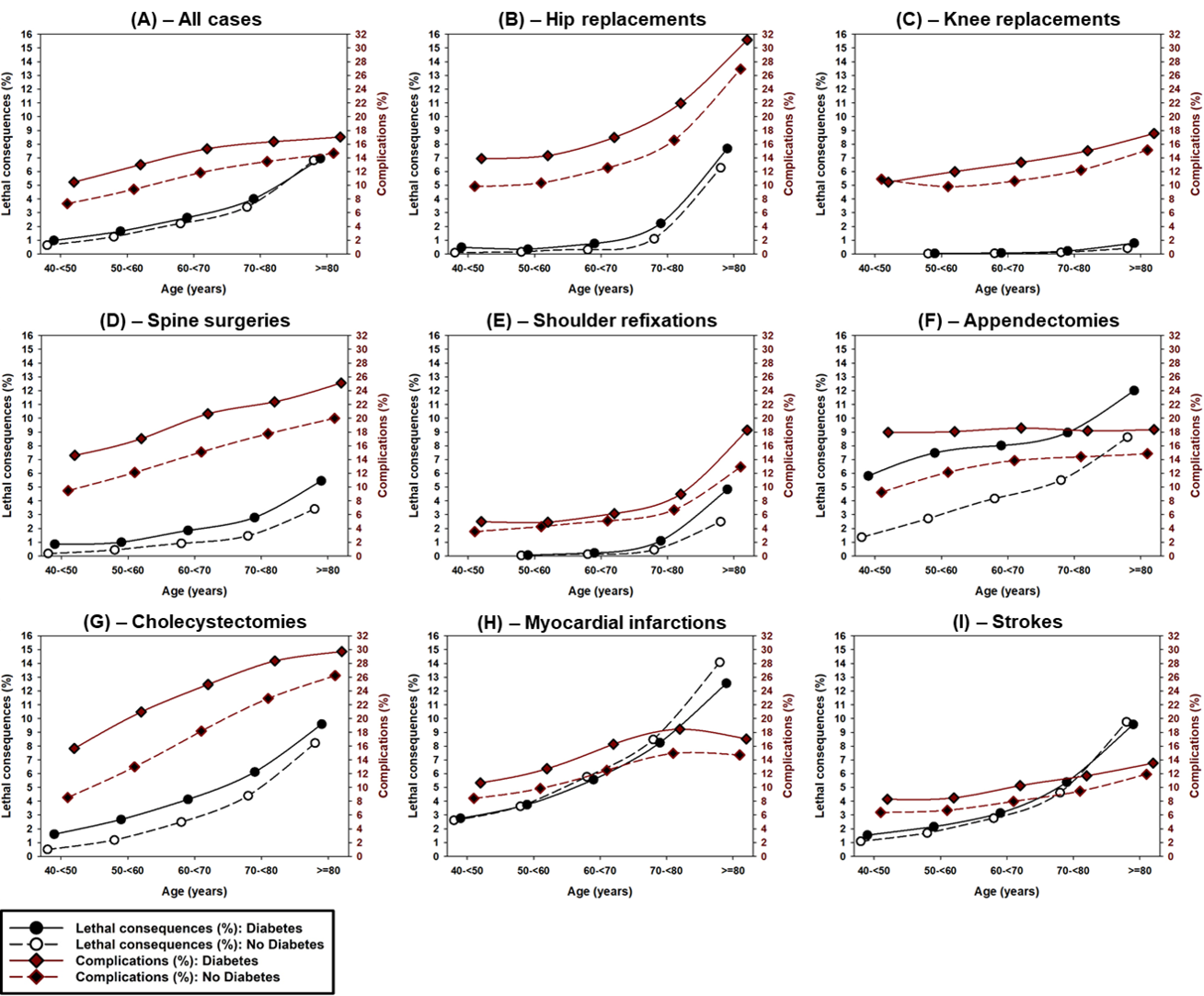

Supplement: Supplementary file 4 — Supplementary figure 3. Complication and mortality rates of all hospitalizations (A) and several procedures and diagnoses (B)–(I) among all hospitalized men with and without diabetes in Germany from 2015 to 2019. [file 508_2023_2153_MOESM4_ESM.tif]

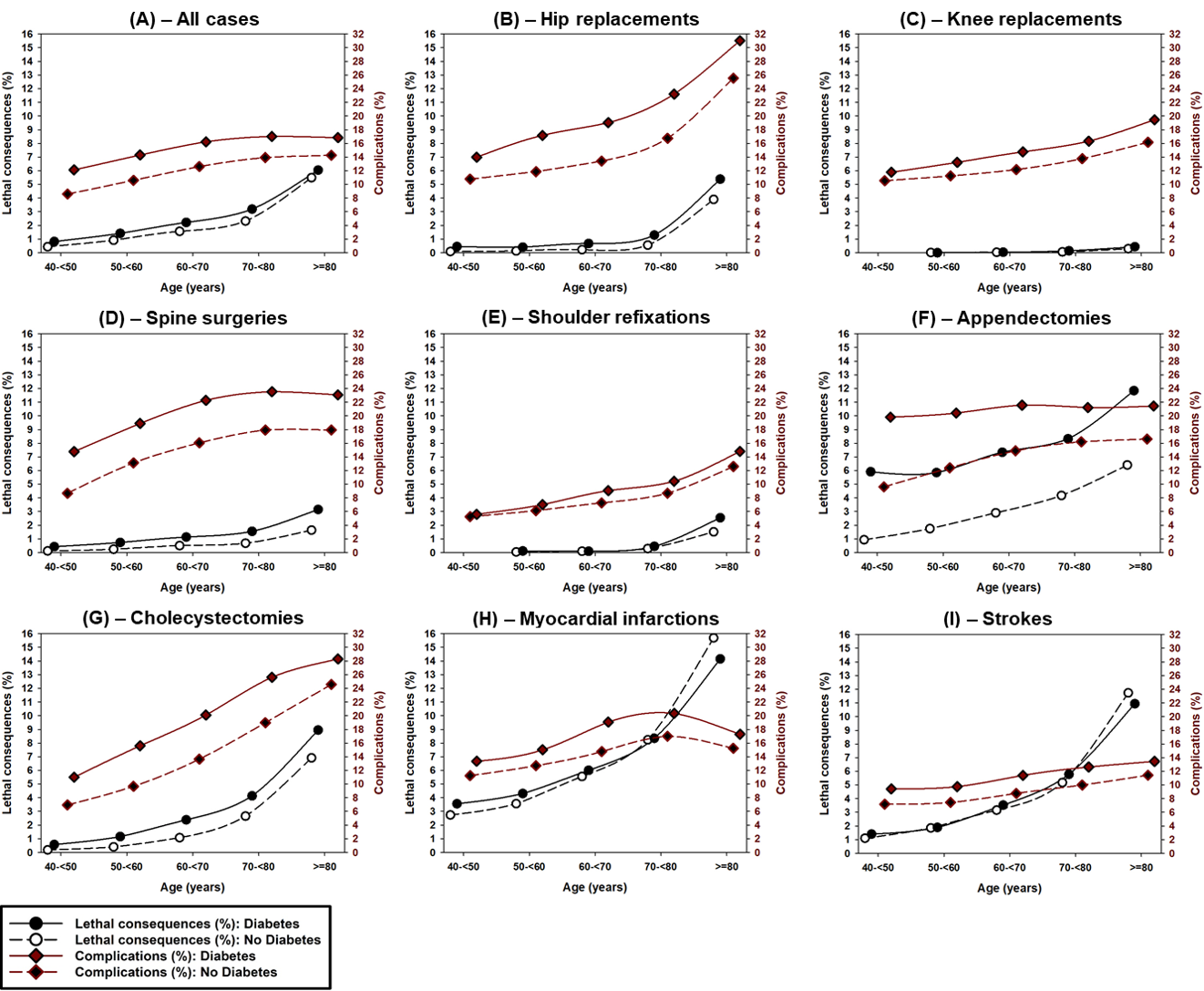

Supplement: Supplementary file 5 — Supplementary figure 4. Complication and mortality rates of all hospitalizations (A) and several procedures and diagnoses (B)–(I) among all hospitalized women with and without diabetes in Germany from 2015 to 2019. [file 508_2023_2153_MOESM5_ESM.tif]
